# Identification of drugs as single agents or in combination to prevent carcinoma dissemination in a microfluidic 3D environment

**DOI:** 10.18632/oncotarget.5464

**Published:** 2015-10-08

**Authors:** Jing Bai, Ting-Yuan Tu, Choong Kim, Jean Paul Thiery, Roger D. Kamm

**Affiliations:** ^1^ BioSystems and Micromechanics IRG, Singapore-MIT Alliance for Research and Technology, 138602, Singapore; ^2^ Department of Mechanical and Department of Biological Engineering, Massachusetts Institute of Technology, Cambridge, MA, 02139, USA; ^3^ School of Mechanical and Automotive Engineering, Kyungil University, Gyeongbuk, 712-701, South Korea; ^4^ Institute of Molecular and Cell Biology, A*STAR (Agency for Science, Technology and Research), 138673, Singapore; ^5^ Department of Biochemistry, National University of Singapore, 117597, Singapore

**Keywords:** epithelial-mesenchymal transition, microfluidics, drug screening, synergistic effect, bladder cancer

## Abstract

Experiments were performed in a modified microfluidic platform recapitulating part of the *in vivo* tumor microenvironment by co-culturing carcinoma cell aggregates embedded in a three-dimensional (3D) collagen scaffold with human umbilical vein endothelial cells (HUVECs). HUVECs were seeded in one channel of the device to initiate vessel-like structures *in vitro* prior to introducing the aggregates. The lung adenocarcinoma cell line A549 and the bladder carcinoma cell line T24 were tested. Dose-response assays of four drugs known to interfere with Epithelial Mesenchymal Transition (EMT) signaling pathways were quantified using relative dispersion as a metric of EMT progression. The presence of HUVECs in one channel induces cell dispersal in A549 which then can be inhibited by each of the four drugs. Complete inhibition of T24 aggregate dispersal, however, is not achieved with any single agent, although partial inhibition was observed with 10 μM of the Src inhibitor, AZD-0530. Almost complete inhibition of T24 dispersal in monoculture was achieved only when the four drugs were added in combination, each at 10 μM concentration. Coculture of T24 with HUVECs forfeits the almost-complete inhibition. The enhanced dispersal observed in the presence of HUVECs is a consequence of secretion of growth factors, including HGF and FGF-2, by endothelial cells. This 3D microfluidic co-culture platform provides an *in vivo*-like surrogate for anti-invasive and anti-metastatic drug screening. It will be particularly useful for defining combination therapies for aggressive tumors such as invasive bladder carcinoma.

## INTRODUCTION

The death of most cancer patients is attributed to metastasis [1]. Micro-metastasis often remains after conventional surgery, radiotherapy and/or chemotherapy, and attempts to treat metastatic cancers have been generally unsuccessful thus far leading to only slightly slowed progression and increased overall survival. Most cancers originate from epithelial tissues, and the process of epithelial to mesenchymal transition (EMT) confers cells with migratory and invasive properties [2, 3]. In this process, carcinoma cells lose their cell-cell junctions and gain an invasive, fibroblast-like morphology, delaminate from the tumor, and intravasate into blood and lymph vessels. Following decades of research, EMT is believed to be a focal event involved in the progression of carcinoma, as well as in generating cancer-initiating cells and in inducing drug resistance [4, 5]. A new anti-metastatic strategy based on inhibiting EMT could impact survival.

Complex networks orchestrate EMT, including activation of a plethora of surface receptors by growth factors, cytokines and extracellular matrix (ECM) components [3, 6–8]. Some of these pathways cooperate to induce EMT with tumor stromal cells, such as endothelial cells, fibroblasts, and tumor-associated macrophages [9]. Existing *in vitro* EMT models (e.g., Transwell™ technology) are also capable of endothelial–tumor co-culture experiments and are achieved by placing endothelial cells on the upper surface of the membrane in close proximity to a lower layer of tumor cells or matrix-containing tumor spheroids to form a three-dimensional (3D) system. However, this technology does not adequately address the topology of the tumor components. In addition, it impairs real-time imaging, rendering tracking of individual cells difficult. Thus, there exists an urgent need to develop more suitable *in vitro* 3D assays that can recapitulate the tumor microenvironment.

Microfluidic assays have been employed in various applications to make *in vitro* assays more realistic, replicating angiogenesis, some of the aspects of organ function, and tumor-endothelial interactions; they have also been used for biopsy studies [10]. By integrating complex environmental factors with assays and on-chip co-culture, this technique controls the 3D microenvironment and enables real-time imaging. Our previous work has demonstrated an intermediate drug assay model capable of monitoring the inhibition of cancer cells migrating away from the primary tumor in 3D culture [11]. This system integrated tumor aggregates in a 3D hydrogel scaffold in close proximity to an endothelial monolayer for screening therapeutic EMT blocking agents. This previous study demonstrated the potential of the microfluidic concept to identify inhibitors of lung adenocarcinoma A549 aggregate dispersal, which is known to be easily reversible from a mesenchymal to an epithelial phenotype.

The current study, in addition to extending the analysis on A549 carcinoma aggregates, seeks to identify drugs that, in combination, could abrogate dispersal of a highly invasive bladder carcinoma cell line. Bladder carcinoma, which becomes life-threatening upon conversion from a superficial to an invasive phase, has yet to truly benefit from the advancements in therapeutic interventions, with the exception of the use of attenuated Bacillus Calmette-Guérin (BCG) intravesical instillation for superficial tumors. Unfortunately, the transition to refractory invasive tumors is almost inevitable. Thus, here we undertook a microfluidics approach to screen for large panels of drug in combination. Employing an improved two-gel system, we performed dose-response assays of four potential drug candidates using the bladder carcinoma T24 cell line [12]. We show that the drugs were less effective in inhibiting T24 cells than A549 cells. Specifically, human umbilical vein endothelial cells (HUVECs) induced cell dispersion in A549 cells, but this dispersion could be inhibited by each of the four drugs. However, inhibiting the spontaneous dispersal of T24 aggregates proved more difficult. Without HUVECs, higher doses of each drug were required, and only partial inhibition could be achieved at 10 μM concentrations of a Src inhibitor, AZD-0530. Even when the four drugs were used in combination, each at a concentration of 10 μM, dispersal was not completely blocked. In the presence of HUVECs, drug resistance was further enhanced. Combination of the four drugs failed to inhibit T24 aggregate dispersal either alone at 10 μM concentration or in combination. Growth factor tests, ELISA, and neutralized antibody blocking experiments revealed that the enhanced dispersal observed in the presence of HUVECs was due to the secretion of growth factors, including HGF and FGF-2, by endothelial cells. The addition of AZD-0530 did not affect the activities of HGF or FGF-2 in inducing cell dispersal. A previous report used integrin-blocking experiments to show that T24 is sensitive to integrin β1-collagen-induced migration for individual cells [13]. However, in the present study, dispersal from aggregates was found to be largely integrin β1-independent.

## RESULTS

**Figure 1:**
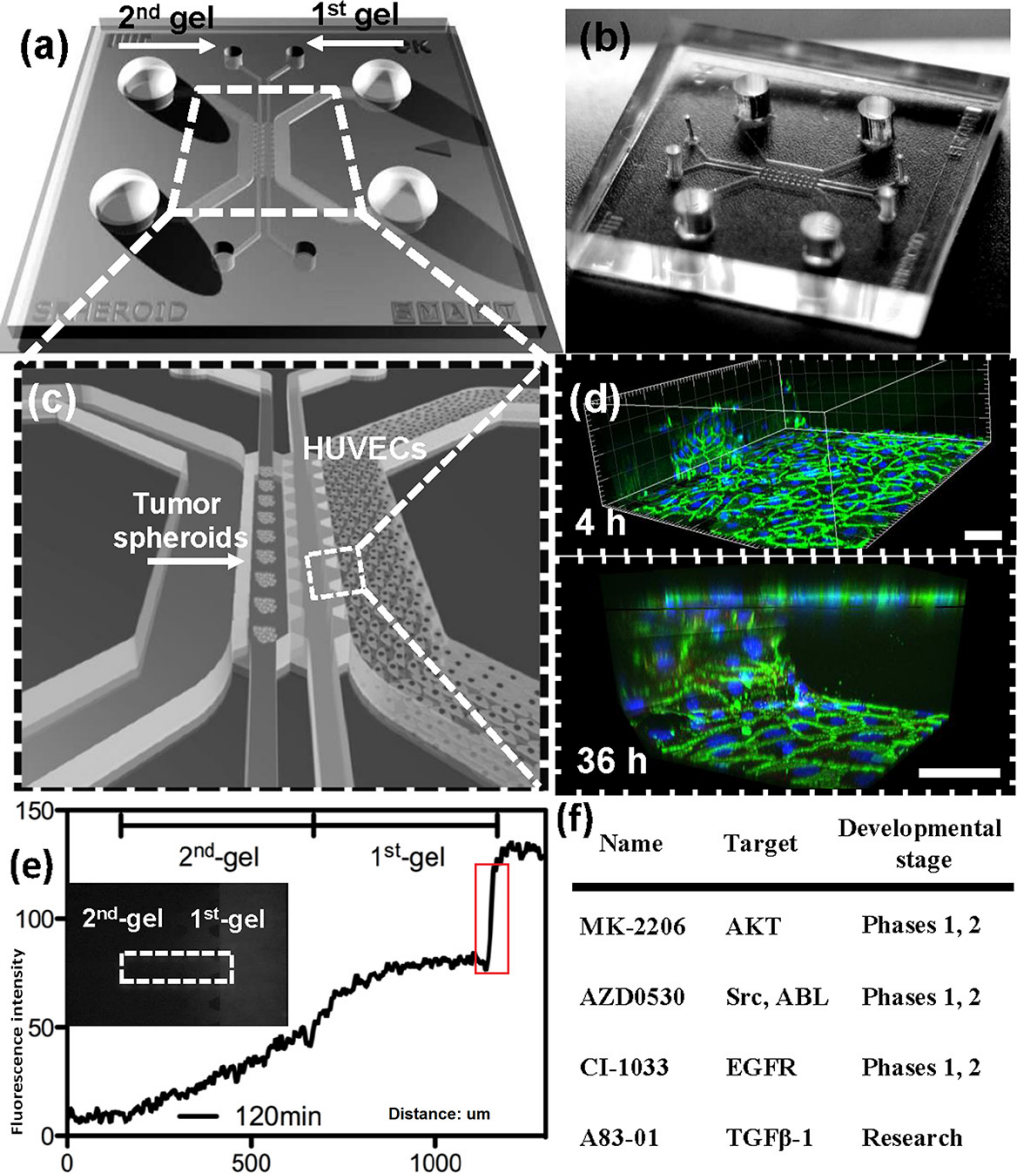
Microfluidic co-culture platform for drug screening **a.** Schematic of device design shows the overall layout of the media channels and gel regions, where the two regions in the middle are used for introducing collagen gel and the two side channels for filling culture medium and growing of endothelial cells. **b.** Photograph of the PDMS device. **c.** An enlarged isometric view of the device showing the relative locations of co-culturing tumor aggregates and endothelial cells (HUVECs). **d.** HUVECs monolayer formed after 4 h and 36 h, respectively, in the microfluidic channel. Green fluorescence is used for VE-cadherin; blue fluorescence indicates DAPI-stained nuclei. **e.** Concentration profiles of fluorescent dextran from which permeability can be quantified. A jump in fluorescence concentration on the right side (circled in red) is due to the presence of the intact endothelial monolayer, thus small molecules can only diffuse through the monolayer, resembling drug diffusing out of a blood vessel. **f.** Drugs used in this study, with the targeting pathways and stage of development.

**Figure 2:**
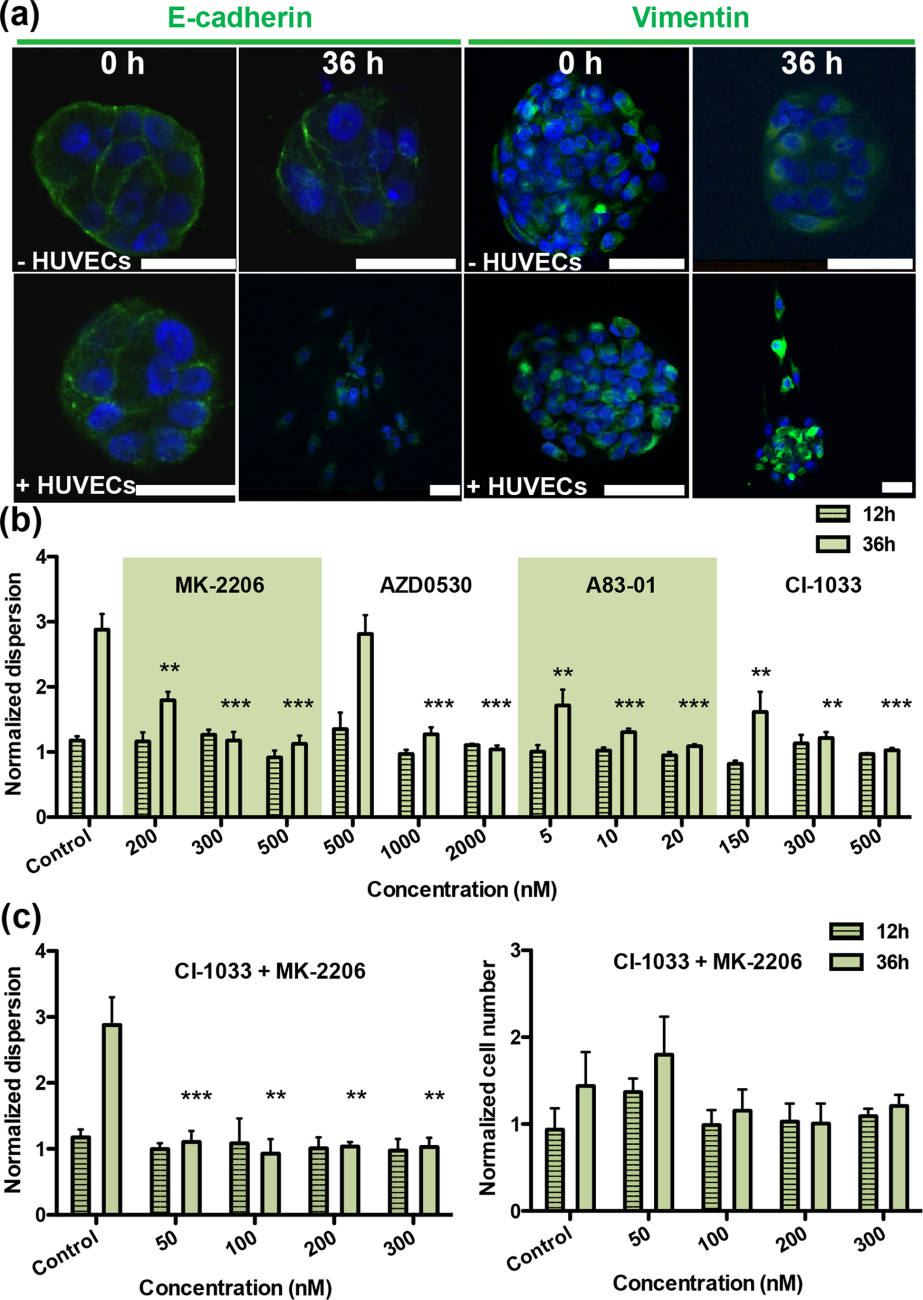
Screening therapeutic drugs on A549 aggregates over 36 h **a.** Staining of EMT markers E-cadherin and vimentin in A549 aggregates at 0 h and 36 h. Green fluorescence is used for both E-cadherin (left panels) and for vimentin (right panels); blue fluorescence is DAPI-stained nuclei. **b.** Normalized dispersion measured for three concentrations with four drugs at 12 h and 36 h (MK-2206: Akt inhibitor; AZD-0530: Src inhibitor; A83–01: TGF-βR inhibitor; CI-1033: EGFR inhibitor), for three different concentrations in the presence of HUVEC. **c.** Normalized dispersion measured over time (12 h and 36 h) for analysis synergistic effects between CI-1033 and MK-2206, at four different concentrations (left: dispersion; right: cell number).

**Figure 3:**
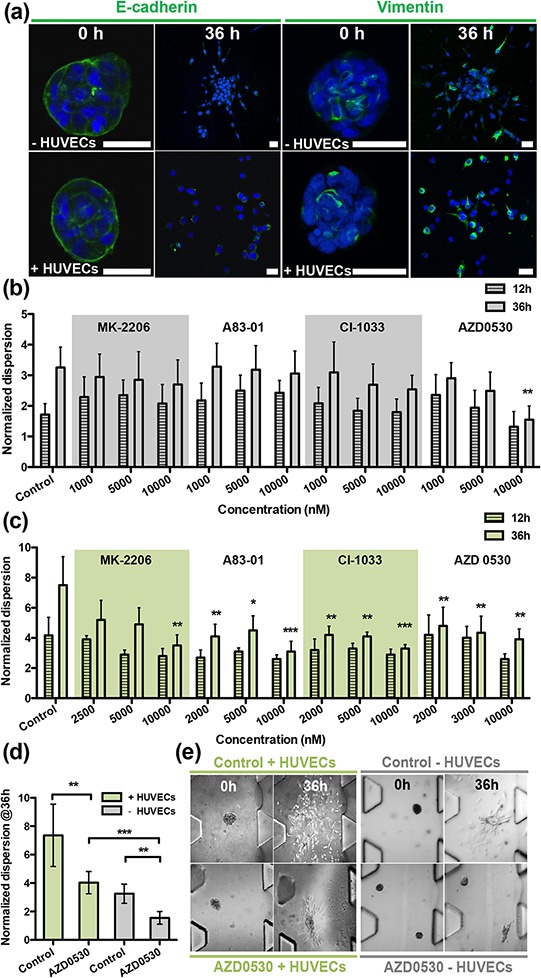
Screening therapeutic drugs on T24 aggregates over 36 h in the presence/absence of HUVECs **a.** Staining of EMT markers E-cadherin and vimentin in T24 aggregates at 0 h and 36 h. Green fluorescence is used for both E-cadherin (left panels) and for vimentin (right panels); blue fluorescence is DAPI-stained nuclei. **b.** Normalized dispersion of T24 cells with four drugs, in the absence of HUVECs, at 12 h and 36 h, for three different concentrations. AZD-0530 is effective at 10,000 nM. **c.** Normalized dispersion of T24 cells with four drugs, in the presence of HUVECs, at 12 h and 36 h, for three different concentrations. AZD-0530 is not effective at 10,000 nM. **d.** Summary of normalized dispersion between control and AZD-0530 (10 μM), treated group, in the presence/absence of HUVECs at 36 h. **e.** Qualitative images of (d).

A549 and T24 cell lines were selected for this study to investigate EMT and tumor progression, since they exhibit a reversible EMT phenotype that could potentially be blocked to achieve EMT reversal. For this study, a microfluidic system (Figure 1(a-c),Supplementary Figure S1) was used, similar in design to a previously reported system [11] except that it incorporated two different 3D collagen compartments between the two media channels. The second gel region was added in order to permit the formation of a more uniform endothelial monolayer. In particular, we have now allowed endothelial cells to form a blood vessel. The carcinoma cell aggregates are introduced in the compartment distal to the channel in which the endothelial cells had assembled to mimic the vascular wall. The endothelial monolayer structure exhibited clear cell-cell junctions, as evidenced by VE-cadherin staining (Figure 1(d)). Integrity of this HUVEC monolayer was also quantified by testing the permeability to 70-kDa dextran rhodamine (Figure 1(e)). The significant drop in fluorescence intensity at the boundary of the first gel (where the HUVEC monolayer resides and highlighted in red rectangle) indicated the formation of an intact monolayer for the drug diffusive microenvironment. Dispersion was quantified and a positive correlation between EMT marker expression and a normalized dispersion of aggregates was observed (see Figure 2(a), 3(a)).

To test the microfluidic drug screening platform for cancer-type specificity, A549 and T24 aggregates were formed in non-adhesive, laser-generated microwells and cultured in the devices. Results for the A549 cells demonstrated that the aggregates do not disperse when cultured in the absence of HUVECs, consistent with our previous study [11]. In the presence of HUVECs, aggregates did disperse, although each of the drugs—MK-2206, AZD-0530, A83–01 and CI-1033 (Figure 1(f)) —could fully suppress dispersal, each at a particular dose (Figure 2(b)-dispersion,Supplementary Figure S2. 1-cell number) within the range of concentrations tested. Thus, we next tested combinations of these same drugs. Interestingly, the EGFR inhibitor MK-2206, CI-1033, and an AKT inhibitor showed a significant synergistic effect, in that the amount of drug required for inhibition of dispersion was reduced to one-fifth that required for each drug individually. Inhibition of aggregate dispersion was reduced from 500 nM to less than 100 nM for each (Figure 2(c)-dispersion, Supplementary Figure 2(d)-cell number). Isobologram for synergistic effects between CI-1033 and MK-2206 is shown in Supplementary Figure S2.2.

**Figure 4:**
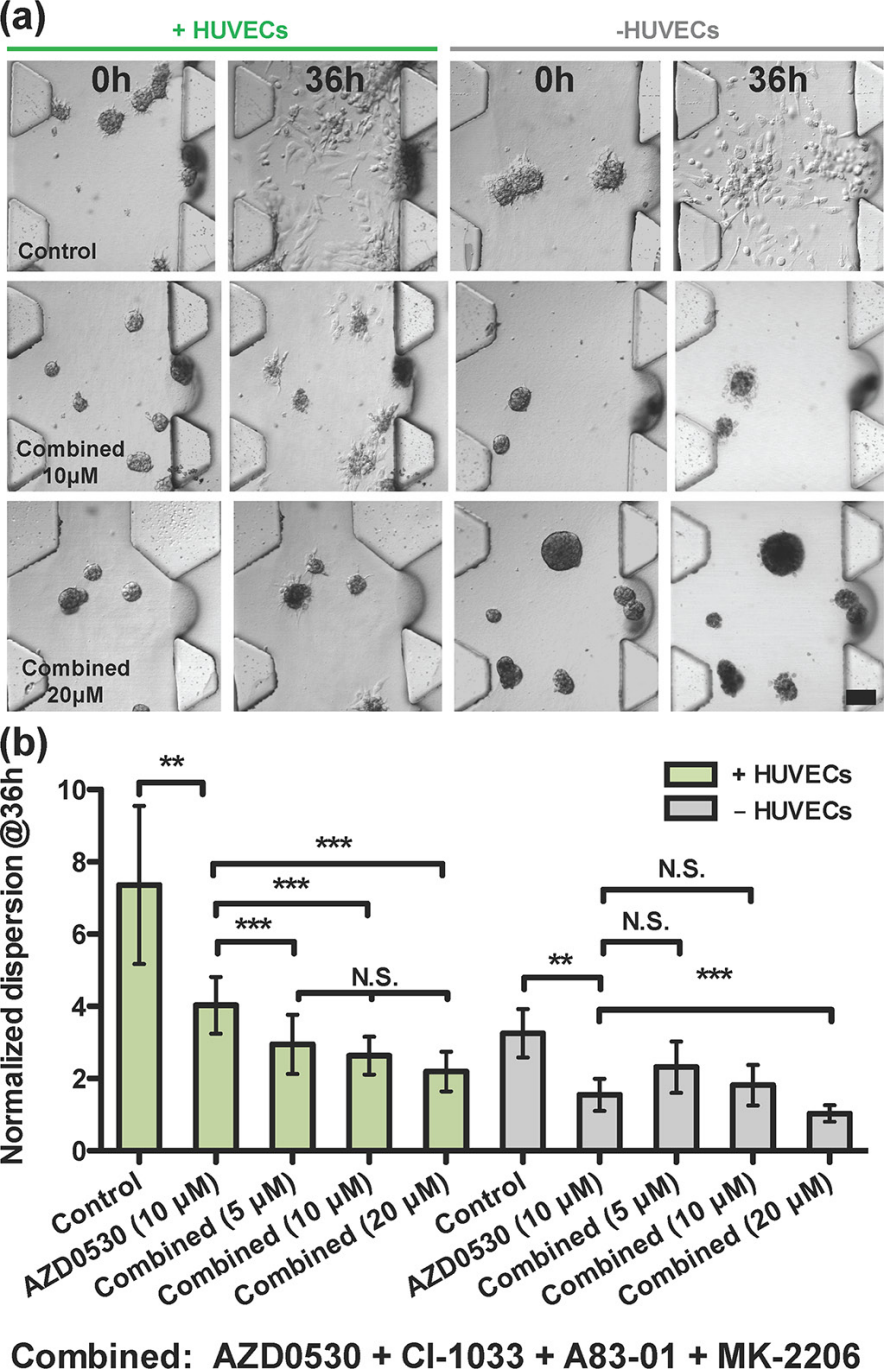
Drug combination analysis on T24 cell aggregates **a.** Qualitative images showing the effect of drug used in combinations of four, in the presence or absence of HUVECs at various doses, 10 μM and 20 μM at 0 h and 36 h. **b.** Comparison between AZD-0530 (used alone at 10 μM) and drugs in combinations of four, in the presence or absence of HUVECs at various doses. Concentrations given are for each drug individually (e.g. combined 5 μM means each drug used at a concentration of 5 μM)

Compared with A549 cells, the dispersion of T24 aggregates alone or in the absence of HUVECs could not be suppressed by any of the four drugs; albeit, a partial inhibition was observed using the Src inhibitor, AZD-0530, at 10 μM (Figure 3(b)-dispersion,Supplementary Figure S3-cell number). Only when the four drugs were added in combination, each at 10 μM concentration (Figure 4(a)-morphology (−HUVEC), (b)-dispersion (−HUVEC)) was almost complete inhibition obtained, which could be considered an additive, rather than synergistic effect. The results indicated that, among the four drugs tested, AZD-0530 is the most effective in preventing spontaneous dispersion of the T24 aggregates. In the presence of HUVECs, each drug had some inhibitory activity on aggregate dissociation; albeit, individually, they were each unable to reduce the dispersion value to less than 3 (complete inhibition corresponds to *a* value of 1). Even AZD-0530 was unable to inhibit dispersal when aggregates were co-cultured with HUVECs (Figure 3(c)). A summary of the AZD-0530 effect in both absence and presence of HUVEC is shown in Supplementary Figure S3(d)-dispersion, S3(e)-morphology.

It is worthwhile noting that the spontaneous dispersion of T24 aggregates alone, without co-culture, is about 3, similar to the value observed in co-culture with HUVECs when maximally inhibited by individual drugs. Even when used in combination, complete inhibition of T24 cancer aggregates dispersal could not be achieved with a combination of any two drugs (Supplementary Figure S4), or when all four drugs were used in combination at 10 μM each (Figure 4(a)-morphology (+HUVEC), 4(b)-dispersion (+HUVEC)), albeit a slight improvement (dispersion reduced to < 2) was observed for all four drugs compared to any single drug or any combination of two drugs (Supplementary Table S1).

**Figure 5:**
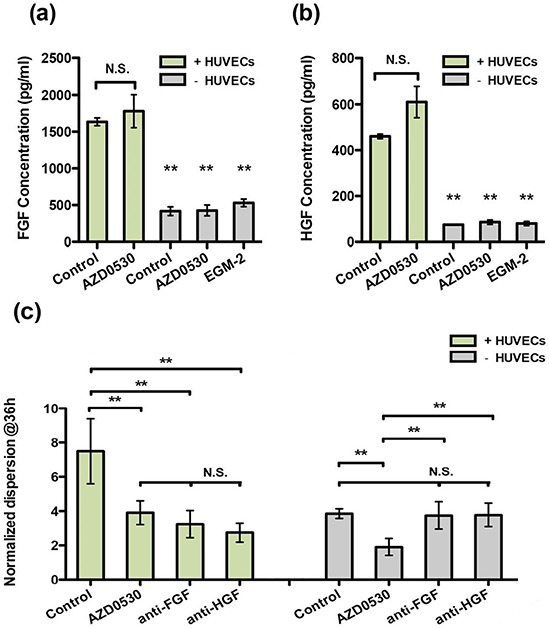
Analysis of endothelial cell secretion of HGF and FGF-2 in co-culture or by T24 cells alone (AZD-0530 was used alone at 10 μM, EGM-2 is the medium control) **a.** ELISA measurement of FGF-2 concentration. **b.** ELISA measurement of HGF concentration. **c.** Neutralized antibody blocking experiment (HGF and FGF-2).

It is likely that some factor(s) — other than those targeted by the drugs listed — are secreted by HUVECs and induce dispersal, bypassing Src signaling. Thus, we next investigated a group of EMT-inducing growth factors, including TGF-β1, TGF-β3, EGF, FGF-2, HGF and PDGF. These factors are possibly secreted by endothelial cells to mediate EMT. In the presence of AZD-0530, HGF and FGF-2-induced aggregate dispersal was reduced to a level comparable with that obtained in co-culture with HUVECs, whereas TGF-β3 and EGF exhibited less of an effect, plus TGF-β1 and PDGF had little effect (Supplementary Figure S5.1). To analyze whether endothelial cells might be inducing EMT in T24 cells through secretion of HGF/FGF-2, we first measured HGF/FGF-2 secretion by HUVECs using ELISA. Concentrations of FGF-2 in co-culture were found to be three times higher than in cultures with aggregates only (Figure 5(a)). Similarly, endothelial cell secretion of HGF was 5-fold higher in co-culture conditions as compared with aggregates alone (Figure 5(b)). The presence of AZD-0530 did not affect the concentrations of either growth factor, and the addition of EGM to the aggregates had no significant effect. This suggests that endothelial cells produce HGF and FGF-2 in co-culture conditions, even in the absence of T24 aggregate co-cultures.

In co-culture with HUVECs, T24 aggregate dispersal was reduced to 40% of control when treated with the HGF neutralizing antibody (Figure 5(c)). The inhibition effect is the same as that achieved by each of the individual drugs. Interestingly, in T24 aggregates alone, the antibody against HGF had little effect on inhibiting dispersal, regardless of the presence or absence of AZD-0530. A neutralizing antibody against FGF-2 delivered a similar degree of inhibition to that observed with anti-HGF antibodies. To validate the antibody inhibitory effects, a c-Met inhibitor, JNJ-38877605 (Johnson & Johnson), and an FGFR inhibitor, TKI-258 (Novartis), were applied each at 10 μM. The results are consistent with the antibody blocking experiment of anti-HGF and anti-FGF-2, leading to dispersal values of approximately 3 (Supplementary Figure S5.2).

**Figure 6:**
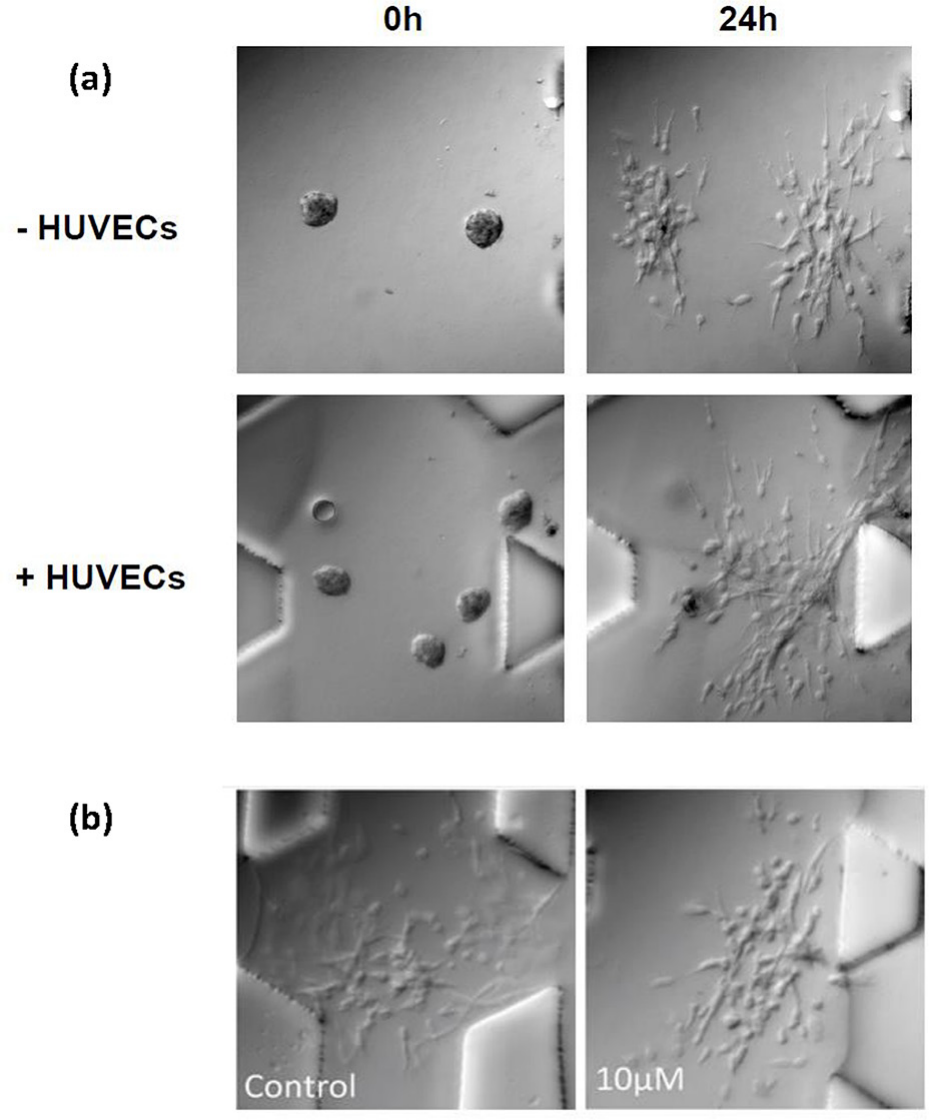
Blocking integrin β1 assay in microfluidic device **a.** Qualitative image for T24 aggregate dispersion with 20 μg/ml β1 antibody, in the absence/presence of HUVECs. **b.** In the presence of HUVECs, qualitative image for T24 aggregate dispersion with 10 μM AZD-0530.

Finally, we investigated the role of integrin β1 on inducing aggregate dispersal via Src signaling. Integrin β1 was blocked by a neutralizing antibody in T24 aggregates in the absence or presence of AZD-0530, both in 2D and 3D conditions. T24 aggregate dispersal could not be inhibited by blocking integrin β1 in 3D, either with aggregates alone or in co-culture conditions that induced dispersion (Figure 6(a)). Even in the presence of 10 μM AZD-0530, blocking of integrin β1 on T24 co-cultured with HUVECs could not prevent dispersion from aggregates (Figure 6(b)). In contrast, it has previously been shown that blocking of integrin β1 in 2D can efficiently inhibit the migration of T24 cells [13]; our results in 2D were consistent with these previous findings (data not shown).

## DISCUSSION

Cancer is a systemic disease occurring in a 3D microenvironment and involves a complex interaction of stromal cells, vascular cells and the tumor, which orchestrate a series of cellular events, often leading to invasion and metastasis. The heterogeneity of various cancer types further complicates these issues, giving rise to the altered efficacy of specific compounds under certain conditions. In this study, we tested drug responses (alone and in combination) on two different cell lines, with a special emphasis on invasive bladder cancer.

Cancer patients usually have elevated serum level of growth factors, namely, epidermal growth factor (EGF), and transforming growth factor-beta (TGF-b). Moreover, there are observed over-expression of tyrosine and serine threonine kinases, e.g. Src, Akt. The kinase oncogenes play essential roles in growth factor signal transduction regulation. Dysregulation of them could cause resistance to cancer cell death and excessive proliferation, in the meantime, initiating and sustaining EMT program, since EMT is modulated by similar signaling pathways for which these molecules have been generated [14]. Small molecule inhibitors against those kinases were developed originally as inhibitors of cell proliferation and have been effective in pre-clinical/clinical trials; these inhibitors were more recently discovered to interfere with EMT, as reported in detailed by Chua, et.al in 2012 [14]. Drugs inhibiting EGFR and Src have also been shown to affect cell motility and invasiveness [15].

By integrating multiple biochemical and biophysical factors and multiple cell types, a microfluidic platform with individual cancer microenvironmental chambers was used to help facilitate an understanding of the crosstalk between cancer and stromal cells. Our microfluidic co-culture with an endothelial cell monolayer and cancer aggregates was able to recapitulate some of the cross-talk that promotes EMT by paracrine interactions between these cell types. A subsequent study from our laboratory will bring in stromal cells, however this study is mandatory to semi-quantitate the behavior of carcinoma cells in 3D collagen in the presence of blood vessels. The integrated system has the potential to be cost-effective and importantly, sensitive with minimum sample usage. Robustness and less likelihood of human error have the additional benefit of reducing the workload.

Our 3D system also revealed important differences between 2D and 3D culture in terms of EMT, and allowed for the real-time visualization and quantification of cell dissemination from aggregates. The two-gel region microfluidic system used in this study was motivated by a need to grow an intact endothelial monolayer prior to embedding the 3D cancer cell aggregates. We found from our previous work that a stabilized, low permeability monolayer was key to reproducing the dynamics of drug delivery and that it offered a better model for the process of drug diffusion across blood/lymph vessel walls into the ECM surrounding the tumor [11]. This combination of 3D co-culture with drug transport across an endothelial monolayer into the matrix surrounding the tumor cell aggregate has the potential for drug screening in a more physiologically-relevant setting, compared to our previous system. Moreover, endothelial derived growth factors are also important constituents in the tumor microenvironment, and their presence in the present experiments further contributes to *in vivo* fidelity. These morphological metrics were supplemented by measurements of epithelial (E-cadherin) and mesenchymal (vimentin) marker expression to assess EMT progression (Figure 2(a)-A549, 3(a)-T24).

In A549 cells, the rapid dissociation of compact cancer aggregates by endothelial cells was reversed by inhibitors of several receptor tyrosine kinases and intracellular kinases (Figure 4). Our data suggest that several pathways were activated in A549 cells by endothelial-secreted growth factors. We also observed a synergistic effect using an EGFR inhibitor (CI-1033) in conjunction with an Akt inhibitor (MK-2206). Previous studies have suggested that this synergistic effect leads to actin cytoskeletal disorganization and, potentially, regulation of Rho GTPases [16]. These findings provide references for the future direction of drug usage in lung adenocarcinoma.

The T24 bladder carcinoma cell line exhibits a mesenchymal-like phenotype, with an EMT score of 0.53 [17] and a more aggressive behavior than lung adenocarcinoma A549, which has an EMT score of 0.28, indicative of more intermediate epithelial-mesenchymal phenotype. In T24 cells, spontaneous dispersal was readily observed in 3D collagen gels. This implies that the two carcinoma cell lines differ significantly in their ability to respond to a collagen-containing ECM, thus influencing their invasive behaviors. Bladder carcinoma cell lines share remarkable invasive properties with bladder tumors, which, *in vivo*, lead to the rapid invasion of the bladder wall. A critical initial step in the development of these approaches is to understand the signaling mechanisms that regulate the tendency for invasion. Changes in the composition and differentiation status of stromal cells accompany the development of malignant cells. Angiogenesis is a critical step at the early stages of tumor progression, which not only contributes to growth of the tumors but, together with other stromal cells, can also promote the dissociation of carcinoma cells. Endothelial cells are recognized as regulators of cancer progression with a synergistic effect adding to the tendency for cancer cells to spontaneously invade the surrounding tissues. Comprehensive analysis of the secretion protein profiles of HUVECs has revealed that multiple paracrine factors could play a role in triggering cancer progression [18].

The presence or absence of endothelial cells also contributes to the differences of drug efficacy in T24 cells. This might explain why some drugs seem to be effective in the earlier screening stage, however, fail in later *in vivo* testing. In this experiment, drugs targeting Akt, EGFR, or TGFβR suppress HUVEC-induced aggregate dispersion from 6–7-fold to ~3-fold, suggesting that endothelial cells produce EGF and members of the TGFβ family. Interestingly, the Src-inhibitor, AZD-0530, was highly effective in inhibiting dispersion in the absence of HUVECs, but this effect was almost completely forfeited when HUVECs were added in co-culture. Both of these results clearly indicate that other factors secreted by endothelial cells also mediate T24 EMT progression whereas Src activation is not essential. Since AZD-0530 is a dual-kinase inhibitor, we also investigated whether inhibition of c-abl kinase also leads to the partial inhibition of T24 aggregate dispersion, by treating with 10 μM of STI-571 (imatinib, data not shown); the results of which suggested that c-abl has little or no role in dispersion.

To investigate the role of paracrine signaling by HUVECs to T24 cells, individual growth factors were examined in the absence of HUVECs and in the presence of AZD-0530. Growth factor test results revealed that either HGF or FGF-2 can restore aggregate dispersal. In this regard, several reports have indicated that HGF/c-Met pathway activation correlates with bladder cancer progression [19–21]. Moreover, muscle invasiveness of T24 cells is associated with high levels of HGF in the serum and urine [22] [23]. Our study shows that HUVECs secrete HGF, which promotes dispersion of T24 cells by a mechanism independent of the Src pathway. ELISA and neutralizing antibody experiments further validated these findings. We also observed that FGF-2 plays a similar role to HGF in promoting EMT. FGF-2 is secreted by HUVECs and was shown to trigger T24 cell dispersal. Previous literature has reported that FGF-2 could lead to EMT through Ras-MAPK pathway activation [24, 25]. In addition, c-Met and FGFR inhibitor experiments produced results that are consistent with our blocking antibody observations. The information in this study is proposed to be useful to provide a framework to guide the selection of the best in class drug combination, to treat invasive bladder carcinoma. It is now envisioned to expand this study by using tumor biopsies rather cell lines to establish new therapeutics regimens.

Our findings regarding Src-mediated T24 aggregate dispersion imply that the inhibition of Src kinase might partially prevent the collagen-triggered EMT of T24 cell aggregates (Figure 6). In type I collagen, β1 integrin is the major cellular receptor [26]. As Src kinase transmits integrin-dependent signaling, and there is evidence that Src is activated at focal adhesions via RhoA, leading to enhanced adhesion disassembly in migrating cells [27, 28], we suspected that integrin β1 might play a role in Src-mediated T24 aggregate dispersion. However, blocking β1-integrin could not prevent the 3D dispersion of T24 aggregates. These results differ from those obtained in a previous study of individual T24 cell migration [29], and suggest that 3D dispersion might rely on different or additional mechanisms as compared with migration that are perhaps associated with membrane metalloproteases [30–32].

## MATERIALS AND METHODS

### Cell maintenance and preparation of carcinoma aggregates

A549 cells transfected with histone H2B-mCherry cDNA and T24 urinary bladder carcinoma epithelial cells were cultured in DMEM (Gibco/Invitrogen 12100) supplemented with 10% FBS (Gibco/Invitrogen). The protocol for generating A549-H2B-mCherry stable cells is described in the Supplemental Data. HUVECs were maintained in microvascular endothelial growth media (Lonza EGM-2MV, Basel, Switzerland). The detailed method for generating cancer cell aggregates is described in our previous study [33, 34].

### Microfluidic device design and fabrication

The microfluidic device for this study was fabricated from polydimethyl siloxane (PDMS) (Figure 1). The PDMS replica is a negative image of the positive relief structure of the patterned wafer made by soft lithography. A detailed description on fabrication of these tissue culture devices can be found elsewhere [35]. The device consists of two gel channels flanked by two media channels that are formed into closed chambers by bonding a coverslip to the PDMS substrate.

The collagen gel solution (2.5 mg/ml, pH = 7.4) was first injected into one of gel channels and polymerized at 37°C to form the endothelial cell adhesion channel. A HUVEC-containing solution was then injected into this channel, and the cells were allowed to form a confluent monolayer on the coverslip bottom substrate and the gel surface. After 24 h, an aggregate-containing collagen gel solution was then pipetted into the second gel region to allow gel polymerization via thermal cross-linking. The average distance between the HUVEC and tumor aggregates was < 200 μm, facilitating rapid cell–cell signaling. Aggregate or individual cell behavior was observed via 3D confocal imaging of the gel region through the supporting glass coverslip. A detailed description of reagent preparation can be found in the Supplemental Data.

### Characterization of endothelial monolayer

Fluorescent dextran 70-kDa Texas Red (Invitrogen) was mixed with culture medium at a concentration of 12.5 μg/ml. The solution was added to the endothelial cell channel, after 24 h of endothelial cell seeding without cancer cell aggregates. Using fluorescence microscopy, the concentration fields were captured at different time points, and their raw intensity profiles were analyzed using MATLAB (Mathworks, Boston, MA, USA).

### 3D drug screening

Four kinase inhibitors regulating EMT on different targets (Figure 1(f)) were selected based on our previous study with an appropriate range of concentrations using A549 and T24 cells, and introduced via the HUVEC channel. For each of the two cell lines, mixtures of two of the four drugs were combined in identical concentrations and tested for drug synergistic rather than additive effects. (Every drug stock solution is prepared at 10 mM in DMSO. The final DMSO concentration in cell culture is < 0.4%). The combination of all four drugs was tested on T24 aggregates. 3D image stacks (a range of 100 μm) of each individual aggregate were acquired by Fluoview 1000 confocal microscopy (Olympus, Japan) with a 20× objective (NA = 0.4). The images were taken at 0, 12 and 36 h. Dispersion was quantified by Imaris 6.0 software (Bitplane). Calculation of aggregate dispersion is described in Supplemental Data.

### Immunofluorescent staining and ELISA

A detailed description of the immunofluorescence staining and ELISA can be found in the Supplemental Data. The antibodies were VE-cadherin (1:100, mouse; Sigma-Aldrich), E-cadherin (1:100, mouse; Sigma-Aldrich) and vimentin (1:200, mouse; Invitrogen). The secondary antibody used was Alexa Fluor 488-conjugated anti-mouse IgG antibodies (Invitrogen). Fluorescent images were obtained using confocal microscopy. ELISA kits were purchased from R&D Systems (HGF: DY294; FGF-2: DY233).

### Blocking experiments

HGF was blocked with 20 μg/ml anti-HGF (AB-294-NA, R&D Systems), FGF-2 was blocked with 5 μg/ml anti-FGF (AB-233-NA, R&D Systems) and integrin β1 was blocked with 20 μg/ml anti-integrin β1 (MAB17781, R&D Systems), respectively. The blocking antibody was added in excess to the experimental setup. In brief, cell aggregates were collected from microwells and blocked for 4 h before mixing with collagen solution and injecting into the microfluidic device. Antibody was continuously supplied via cell media in microfluidic channel, and media were changed on a twice-daily basis. Aggregate dispersion was evaluated at 0 and 36 h.

## CONCLUSION

This study employs a microfluidic-based platform, integrating quantitative drug screening to interfere with mechanisms driving invasion and micro-metastasis induced by the cross-talk between invasive cancer cells and the microenvironment. We demonstrate the utility of this assay in assessing tumor-specific therapeutic potential, by determining the efficacy of drugs as single agents or in combination; this will be particularly important when dealing with highly invasive cancers. The newly uncovered therapeutic regimens could then be considered for further *in vivo* investigations. Our laboratory is currently testing such drug cocktails in bladder preclinical models. In addition, by reproducing EMT in an *in vitro* model using a 3D collagen scaffold with various types of cancer cells, we are able to investigate the tumorigenicity and invasiveness of organ-specific cancers, with potential applications toward the future goal of personalized medicine.

## SUPPLEMENTARY FIGURES AND TABLE


