# Supplementary material for: Identification of drugs as single agents or in combination to prevent carcinoma dissemination in a microfluidic 3D environment
Source: Oncotarget. 2015 Oct 8;6(34):36603–14. doi: 10.18632/oncotarget.5464 (PMC4742198; doi:10.18632/oncotarget.5464)
Supplement: Supplementary file 1 [file oncotarget-06-36603-s001.pdf]

SUPPLEMENTARY METHODS

Generation of A549-H2B-mCherry stable cells

Human lung carcinoma cell line A549 was obtained from ATCC (CCL-185). PH2B_mCherry_IRES_puro2 plasmid was obtained from Addgene (Addgene plasmid 21045). A549 cells were transfected with FuGENE 6 (Roche) and positively transfected cells (red) were treated with puromycin (BD Science, USA). Cells surviving after puromycin treatment were selected by FACS sorting and maintained to generate stable H2B-mCherry expressing A549 cells.

Preparation of reagents for endothelial monolayer

200 µl collagen gel solution (2.5 mg/ml and pH 7.4) was prepared with 105.7 µl type I collagen (4.73 mg/ml, BD Biosciences), 20 µl PBS (10x) with phenol red (Gibco/Invitrogen, USA), 68.3 µl deionized water, 6 µl NaOH (0.5 N). The solution containing cellular aggregate was prepared at 2.5 µg/ml and pH 7.4 on ice with 105.7 µl type I collagen (4.73 mg/ml, BD Biosciences), 20 µl PBS (10x) with phenol red (Gibco/Invitrogen, USA), 48.3 µl deionized water, 6 µl NaOH (0.5 N), and mixed with 20 µl cell suspension medium containing 10–20 tumor aggregates. After formation of endothelial monolayer and injection of cancer aggregate, DMEM and EGM-2 were subsequently introduced to the media channels without and with the endothelial monolayer, respectively.

Calculation of cancer aggregate dispersion

The software determined the number of nuclei for a specific aggregate and generated a histogram of nuclei distribution in the 3D domain. The spheroids centroid location (X_c,Y_c,Z_c) is computed from the coordinates of all nuclei (X_n,Y_n,Z_n). From the locations of all nuclei, the standard deviation from the aggregate center could be termed (Δ, of a given spheroid). Two metrics were used to evaluate drug efficacy: normalized dispersion (Δ/Δ_0), where Δ_0 is calculated from the initial image at $t = 0$, and normalized cell number (N/N_0), where N_0 is calculated from the same initial image at $t = 0$.

$$\Delta = \sqrt{\sigma_x^2 + \sigma_y^2 + \sigma_z^2}$$

Note that the increase in Δ associated with proliferation was assumed to be negligible since the increase in N/N_0 would scale ~ $(\Delta/\Delta_0)^3$.

Immunofluorescent staining and ELISA

Cell culture medium was removed from the microfluidic devices and samples were rinsed in cold PBS and then fixed in 4% paraformaldehyde (PFA) (Sigma-Aldrich, St.Louis, MO, USA) for 15 min at room temperature. Then 0.1% Triton-X (Sigma-Aldrich, St.Louis, MO, USA) was added for permeabilization for 5 min before blocking. Afterwards, the blocked samples were stained with VE-cadherin for demonstration of the endothelial cell monolayer formation; E-cadherin and vimentin were stained separately for evaluation on EMT marker expressions. Nuclei were stained with DAPI (Sigma-Aldrich). The secondary antibody used was Alexa Fluor 488-conjugated anti-mouse IgG antibodies (Invitrogen).

For ELISA, cell culture media from microfluidic devices were extracted after 24 h and placed in wells of a 96-well plate with a two-fold dilution. A 7-point standard curve was drawn for quantification of HGF and FGF, respectively. ELISA data expressed as absorbance at 570 nm on a microplate reader (Benchmark Plus).

SUPPLEMENTARY FIGURES AND TABLE

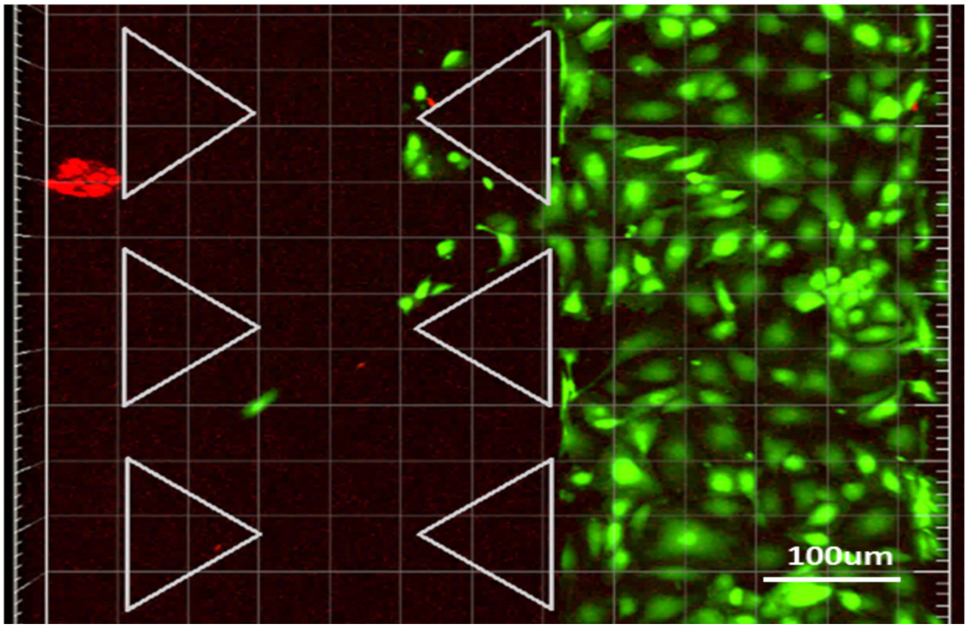

Supplementary Figure S1: (for Figure 1) Microfluidic co-culture platform for drug screening (Fluorescent view) Green fluorescence is GFP-labeled HUVEC monolayer, red fluorescence is A549 aggregate. White triangles are PDMS pillars (artificial).

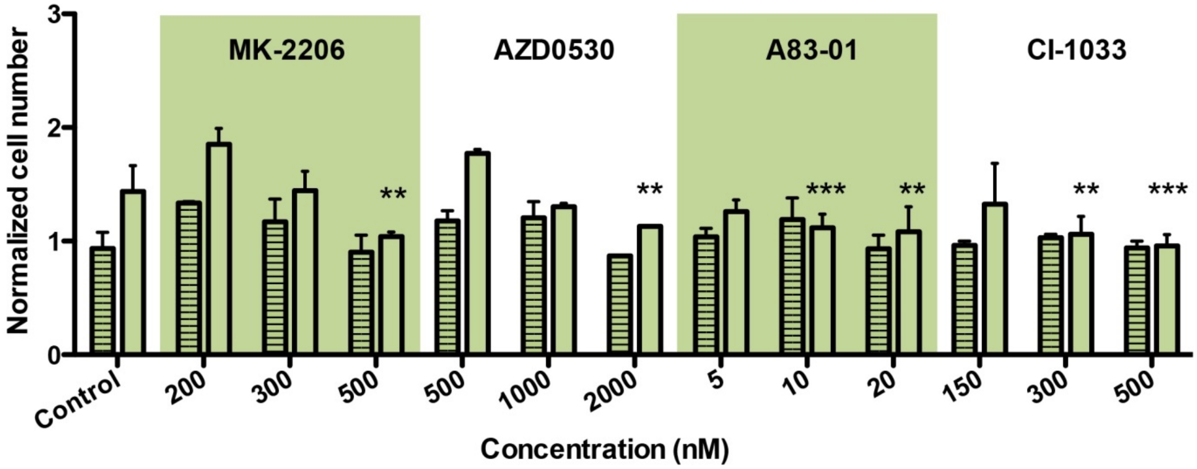

Supplementary Figure S2.1: (for Figure 2) Normalized A549 cell number measured for three levels of concentrations with four drugs (MK-2206: Akt inhibitor; AZD-0530: Src inhibitor; A83–01: TGF-βR inhibitor; CI-1033: EGFR inhibitor).

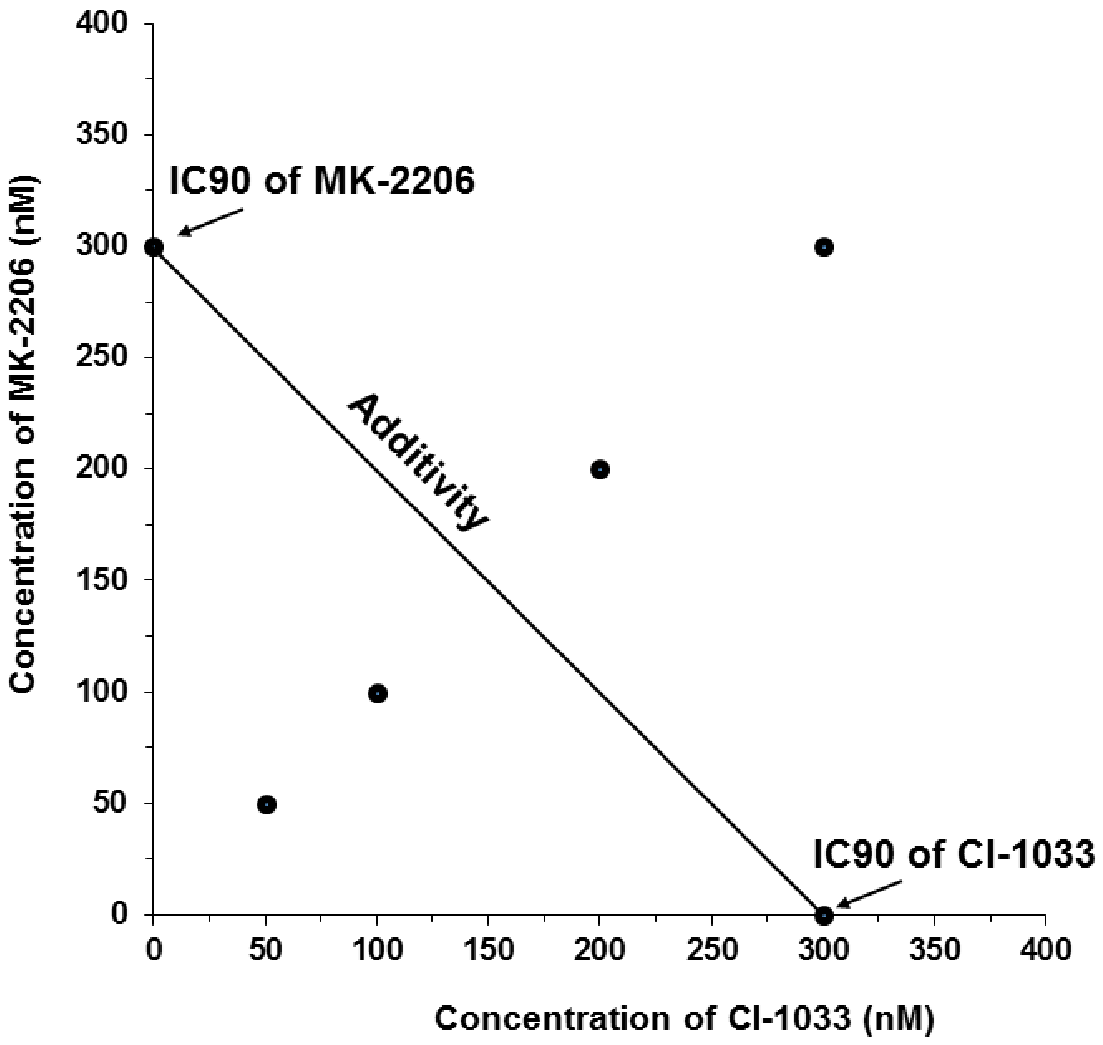

Supplementary Figure S2.2: (for Figure 2) Isobologram for study synergistic effects between CI-1033 and MK-2206, for A549 cell line.

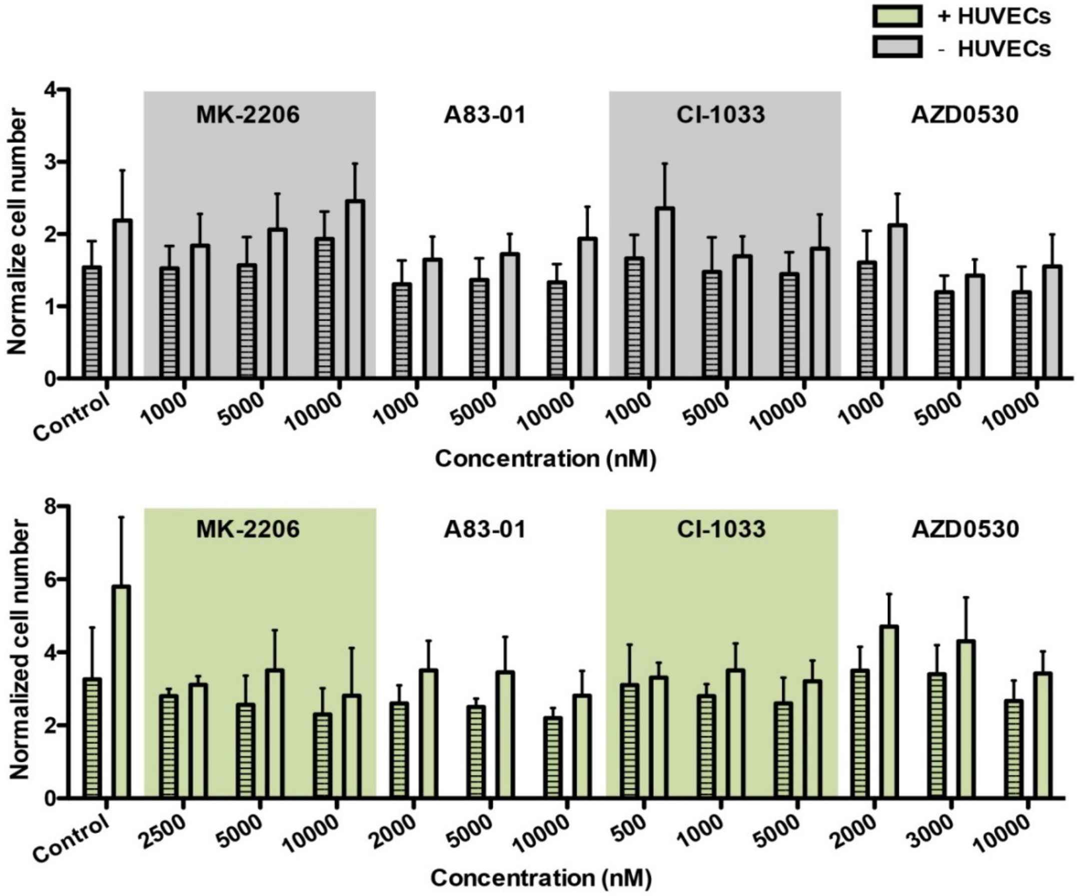

Supplementary Figure S3: (for Figure 3) Normalized T24 cell number measured for three levels of concentrations with four drugs (MK-2206: Akt inhibitor; AZD-0530: Src inhibitor; A83–01: TGF-βR inhibitor; CI-1033: EGFR inhibitor), in the absence/presence of HUVECs.

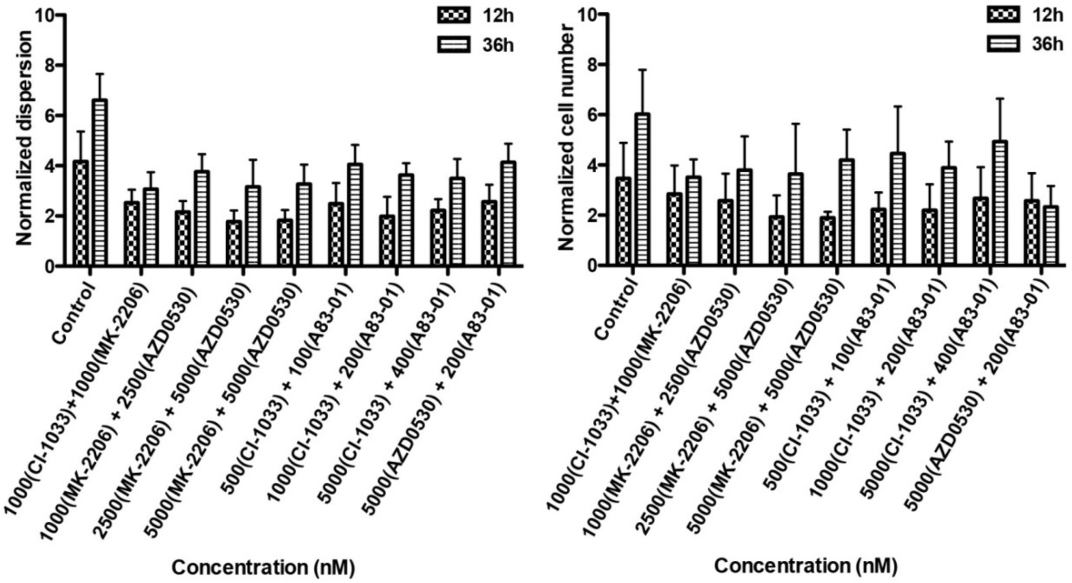

Supplementary Figure S4: (for Figure 4) T24 cellular response to drug synergistic doses. Normalized dispersion and cell numbers measured over time for synergistic effect analysis of four drugs, with every two drugs in combination.

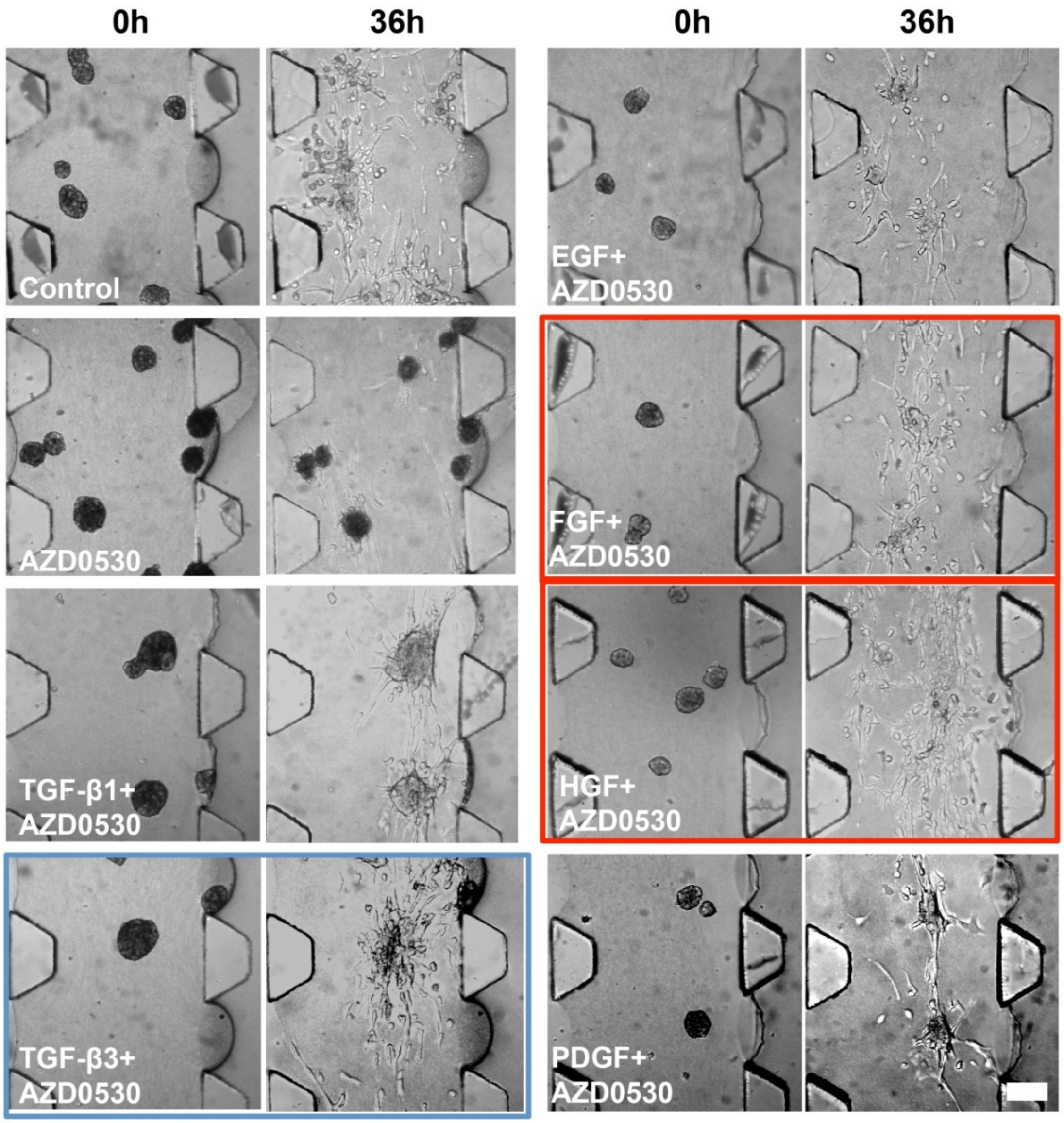

Supplementary Figure S5.1: (for Figure 5) Growth factors testing on triggering cancer aggregate dispersion, in the presence of Src inhibitor (AZD-0530). Qualitative images of various growth factors inducing EMT.

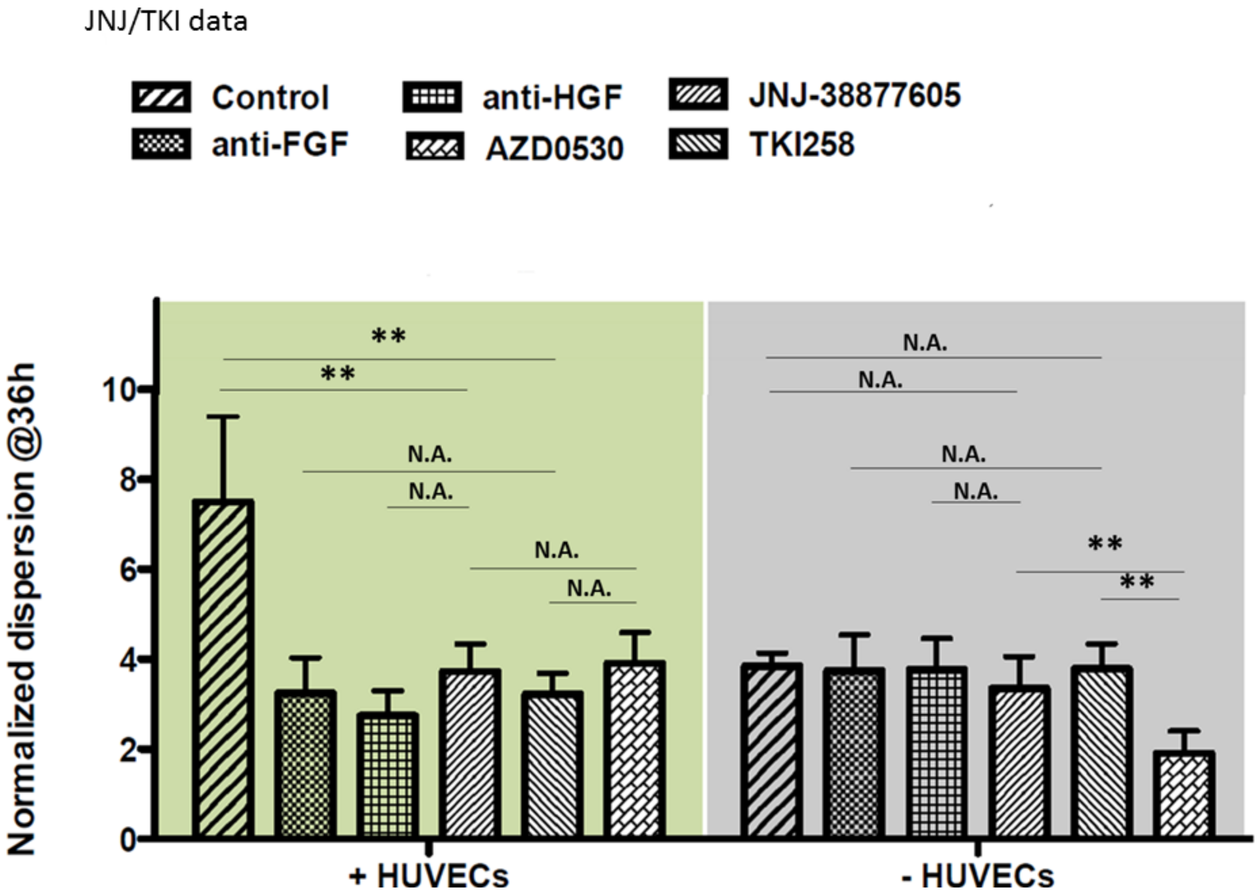

Supplementary Figure S5.2: (for Figure 5(c) extension) A c-Met inhibitor, JNJ-38877605, and a FGFR inhibitor, TKI-258 were applied to T24 aggregate each at 10 µM. Green background is the normalized dispersion in the presence of HUVEC; gray background shows the normalized dispersion in the absence of HUVEC (anti-HGF and anti-FGF are the neutralized ntibodies).

Supplementary Table S1: Table 1: Summary of the drug inhibition results

		A549	T24
No drugs	No ECs	No dispersion	Dispersion
	ECs	Dispersion	Dispersion
Individual drugs	No ECs	-	Partial inhibition by AZD-0530 at 10 µM
	ECs	Full inhibition	No inhibition
Drug used in combination	No ECs	-	Partial inhibition at 10 µM each
	ECs	Full inhibition/ Synergistic effect observed.	No inhibition

(EC = endothelial cell).
